# Utility of the Heart-to-Single Vertebra Ratio Compared with Artificial Intelligence-Derived Vertebral Heart Score for Detecting Cardiomegaly in Dogs with Myxomatous Mitral Valve Degeneration

**DOI:** 10.3390/vetsci13101065

**Published:** 2026-10-09

**Authors:** Berfin Eftelya Güder, Zeynep Nurselin Kot, Viktor Szatmári

**Affiliations:** 1 Department of Internal Medicine, Faculty of Veterinary Medicine, Ondokuz Mayıs University, 55280 Samsun, Türkiye; 2 Department of Clinical Sciences, Faculty of Veterinary Medicine, Utrecht University, Yalelaan 108, 3584 CM Utrecht, The Netherlands

**Keywords:** AI, ACVIM Consensus, B1, B2, HSVR, HSVR-T7, vertebral heart scale, vertebral heart size, VHS, VLAS

## Abstract

The heart-to-single vertebra ratio measured at the seventh thoracic vertebra (HSVR-T7) is a recently described radiographic method used to assess heart size in dogs. Our study evaluated the diagnostic performance of HSVR-T7 for identifying echocardiographically defined cardiomegaly in dogs with myxomatous mitral valve disease (MMVD). Its correlation with vertebral heart score (VHS) and vertebral left atrial size (VLAS), and as its discriminatory performance compared with artificial intelligence (AI)-derived VHS and VLAS measurements were investigated. Radiographs of 93 dogs with echocardiographically confirmed myxomatous mitral valve disease were included; 35 were classified as ACVIM stage B1 and 58 as stage B2 or C. Among the 35 stage B1 dogs, five (14.3%) had isolated left atrial enlargement and two (5.7%) had isolated left ventricular enlargement. The HSVR-T7 scores were significantly higher in dogs with echocardiographically confirmed cardiomegaly (stage B2 or C) than in dogs without cardiomegaly (stage B1): 11.65 ± 1.13 versus 10.47 ± 1.08 (*p* < 0.001). At the optimal cut-off value of ≥11.15, HSVR-T7 yielded an area under the curve (AUC) of 0.769 of the receiver operating characteristic (ROC) curve, with 70.7% sensitivity and 74.3% specificity. The HSVR-T7 showed a very strong positive correlation with VHS (r = 0.890; *p* < 0.001). Artificial intelligence-derived VHS showed the highest diagnostic performance at an optimal cut-off value of ≥11.35 (AUC, 0.861; sensitivity, 75.9%; specificity, 82.9%), whereas AI-derived VLAS yielded an AUC of 0.765, with 62.1% sensitivity and 85.7% specificity at an optimal cut-off value of ≥2.65. Overall, HSVR-T7 was less sensitive and specific in detecting cardiomegaly in dogs with myxomatous mitral valve disease than VHS, and it cannot replace echocardiographic, VHS or VLAS measurements.

## Simple Summary

Myxomatous mitral valve degeneration is the most common heart disease in dogs, and it can cause heart enlargement as the disease progresses. Chest radiographs are commonly used to assess heart size, especially when ultrasound examination of the heart is unavailable. In this study, we evaluated a new, recently described radiographic measurement that determines heart size based on the length of the seventh thoracic vertebra (HSVR-T7). Radiographs of 93 dogs with myxomatous mitral valve disease were included; 58 had heart enlargement and 35 did not based on cardiac ultrasound examination. The tested new measurement (HSVR-T7) resulted in a higher score in dogs with heart enlargement than in dogs with normal-sized heart on ultrasound. The HSVR-T7 measurements were closely correlated to the traditional Vertebral Heart Score (VHS), a commonly used radiographic method for measuring heart size in dogs. The ability of HSVR-T7 to distinguish dogs with and without heart enlargement was similar to that of another radiographic method which is used to assess enlargement of the left upper heart chamber (Vertebral Left Atrial Size). However, our findings suggest that vertebral heart score (VHS) discriminates dogs with and without heart enlargement on chest radiographs better than the HSVR-T7.

## 1. Introduction

Approximately 10% of dogs presented to primary care veterinary practices have a form of heart disease, of which myxomatous mitral valve degeneration (MMVD) is the most common [1]. As mitral valve insufficiency progresses, severe chronic mitral valve regurgitation can cause left atrial and ventricular enlargement, and, in some dogs, it can lead to left-sided congestive heart failure and eventually to cardiac death [1,2,3]. The consensus guidelines of the American College of Veterinary Internal Medicine (ACVIM) indicate that radiographic evidence of cardiomegaly may be used in differentiating dogs with stage B1 (i.e., subclinical disease stage without left atrial and ventricular dilatation) and B2 disease (i.e., subclinical disease stage with left atrial and left ventricular dilatation) when echocardiographic assessment is unavailable [1]. In this setting, vertebral heart score (VHS) ≥11.5 and vertebral left atrial size (VLAS) ≥3.0 may indicate left ventricular and left atrial enlargement, respectively, consistent with stage B2 disease [1]. Although vertebral heart score is useful for detecting generalized cardiomegaly, it may be influenced by factors such as breed, hydration status, and human measurement error, including intra- and interobserver variability [4,5,6,7,8,9]. Artificial intelligence (AI)-derived software has been shown to provide rapid, reproducible, operator-independent, and accurate vertebral heart score measurements [6].

Recently, a new radiographic measurement, the heart-to-single-vertebra ratio (HSVR) was proposed as a valid and reliable alternative to vertebral heart score in dogs [10] and cats [11]. The authors claimed it as a faster method, which might also be used in dogs with vertebral anomalies [10]. Among the various HSVR measurements, the heart-to-single vertebra ratio measured at the seventh thoracic vertebra (HSVR-T7) correlated most closely with the vertebral heart score [10]. This method uses the length of the 7th thoracic vertebral body as a reference [10]. This newly introduced technique demonstrated good-to-excellent interobserver agreement, and provided a simple and rapid method for radiographic assessment of the cardiac silhouette in their study [10].

Our study aimed to evaluate the diagnostic performance of HSVR-T7 for identifying cardiomegaly in dogs with an echocardiographic diagnosis of myxomatous mitral valve degeneration. We hypothesized that HSVR-T7 would correlate positively with AI-derived vertebral heart score and vertebral left atrial size measurements, and it would discriminate between dogs with and without echocardiographically defined left atrial and left ventricular dilatation; in other words, it would distinguish stage B1 from more advanced disease stages, i.e., stages B2 and C. A secondary objective of our study was to evaluate the utility of automated AI-derived vertebral heart score and vertebral left atrial size measurements for distinguishing dogs with cardiomegaly (i.e., stages B2 or C) and without cardiomegaly (i.e., stage B1).

## 2. Materials and Methods

### 2.1. Animals and Echocardiographic Measurements

Dogs with the diagnosis of myxomatous mitral valve degeneration were searched in the electronic database and the physical archive of the Companion Animal Teaching Hospital of the Faculty of Veterinary Medicine of the Utrecht University in the Netherlands, in a period of 16 years, between 2 June 2010 and 27 July 2026. Dogs were only included in this study, if echocardiography and thoracic radiography were performed on the same day at this institution. All echocardiographic and radiographic examinations were performed on awake, non-sedated dogs.

Breed, age, sex, neuter status, body weight, examination date, echocardiographic diagnosis, ACVIM disease stage, and various radiographic cardiac measurements were recorded. If a dog had more than one suitable examination, the first eligible examination was selected. Therefore, each dog was allowed to participate only once in this study.

Echocardiography was performed by a board-certified veterinary cardiologist or a cardiology resident under the direct supervision of a board-certified veterinary cardiologist. For the purposes of this study, the term “cardiomegaly” refers to echocardiographically defined left-sided cardiomegaly, i.e., both the left atrium and the left ventricular diastolic internal chamber dimensions exceeded the cut-off values according to the most recent version of the ACVIM Consensus guidelines, published in 2019 [1]. The left atrial-to-aortic root ratio (LA/Ao) was obtained from a two-dimensional right parasternal short-axis view at the level of the aortic root using the standard (“Swedish”) method as it was described by Dr. Hansson in Uppsala [1]. Measurements were performed in early diastole on the first frame after aortic valve closure. The aortic route diameter was measured with the inner edge to inner edge method along the commissure between the noncoronary and right coronary aortic valve cusps [1]. The left atrial diameter was measured along a line extending from and parallel to the commissure between the noncoronary and left coronary cusps to the distant wall of the left atrium [1]. The LA/Ao ratio was calculated by dividing the left atrial diameter by the aortic diameter (both in mm).

The left ventricular internal dimension at end-diastole (LVIDd) was measured at the onset of the QRS complex using two-dimensional image-guided M-mode echocardiography from the right parasternal short-axis view at the level of the chordae tendineae, using the leading edge to leading edge method. The body-weight-normalized left ventricular internal diameter at end-diastole (LVIDdN) was calculated using the allometric formula as it was described by Cornell et al.: LVIDdN = LVIDd (cm)/[body weight (kg)]^0.^^29^^4^ [1].

Based on the echocardiographic criteria of the ACVIM consensus guidelines, dogs were divided into a cardiomegaly group (*n* = 58; both LA/Ao ≥1.6 and LVIDdN ≥ 1.7; i.e., stage B2 or C) and a non-cardiomegaly group (*n* = 35; i.e., stage B1). According to this operational definition, dogs with subclinical myxomatous mitral valve disease and meeting only one of the two echocardiographic enlargement criteria were retained in the non-cardiomegaly (i.e., stage B1) group. Therefore, isolated left atrial enlargement (LA/Ao ≥ 1.6 and LVIDdN < 1.7) or isolated left ventricular enlargement (LA/Ao < 1.6 and LVIDdN ≥ 1.7) was not considered an exclusion criterion. Dogs with pleural effusion, mediastinal masses, dilated cardiomyopathy, congenital heart disease, primary pericardial disease, cardiac neoplasia, or other cardiac conditions that could cause cardiomegaly independently of myxomatous mitral valve degeneration were not included. Only radiographs of excellent quality were used.

### 2.2. Radiographic Examination

The vertebral heart score and vertebral left atrial size were measured automatically on digital, anonymized, left lateral radiographs using a commercially available deep learning-based AI-based software (MetronMind 4.4.3, Inc., Paso Robles, CA, USA).

**Figure 1:**
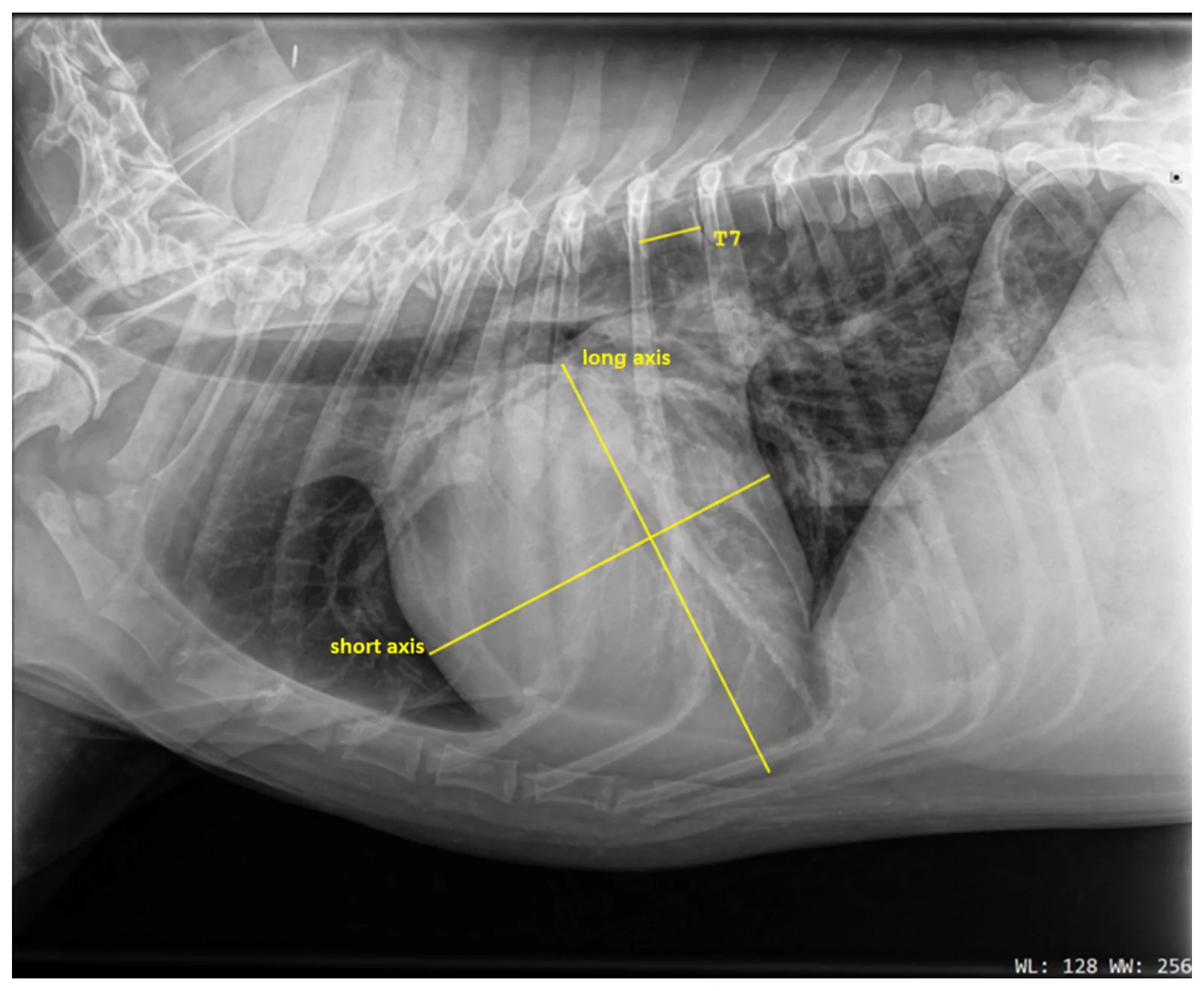
The HSVR-T7 measurement in a dog with echocardiographic left atrial and left ventricular dilatation, and radiographic cardiomegaly. T7 = length of the seventh thoracic vertebral body.

The heart-to-single-vertebra ratio measured at the seventh thoracic vertebra (HSVR-T7) was measured manually by one observer (B.E.G.), who was blinded to the echocardiographic stage of the dogs. The long axis and short axis cardiac dimensions were taken manually from the automatic vertebral heart size measurements, while the seventh thoracic vertebral body (T7) was measured manually by a single observer (B.E.G.). No manual adjustment of the landmarks or measurement axes that were automatically generated by the software was performed in any case. The length of the seventh thoracic vertebral body (T7) was measured using an electronic caliper, including the adjacent caudal intervertebral disc space, according to the original description [10]. The HSVR-T7 value was calculated by dividing the sum of the cardiac axes (long + short axis in millimeters) by the length of the T7 vertebral body (in millimeters) [10] (Figure 1).

### 2.3. Statistical Analysis

Statistical analyses were performed using a commercially available statistical software package (SPSS Statistics, Version 21.0; IBM Corp., Armonk, NY, USA). The normality of continuous variables was assessed using the Kolmogorov–Smirnov and Shapiro–Wilk tests, together with visual inspection of histograms and normal Q–Q plots and evaluation of skewness and kurtosis values. In cases of discordance between the normality tests, the distribution was classified based on the overall assessment of these criteria. Normally distributed variables were compared between dogs with and without echocardiographically defined left-sided cardiomegaly using the independent-sample *t*-test. Non-normally distributed variables were compared using the Mann–Whitney U test.

Linear associations among vertebral heart score, vertebral left atrial size, and HSVR-T7 were assessed using Pearson’s correlation analysis. The strength of the correlations was interpreted according to the absolute value of the correlation coefficient as follows: 0.00–0.19, very weak; 0.20–0.39, weak; 0.40–0.59, moderate; 0.60–0.79, strong; and 0.80–1.00, very strong [12]. Receiver operating characteristic (ROC) curve analysis was performed to evaluate the discriminatory performance of vertebral heart score, vertebral left atrial size, and HSVR-T7 for identifying echocardiographically defined left-sided cardiomegaly. The area under the ROC curve (AUC), together with its 95% confidence interval (CI), as well as sensitivity and specificity with their corresponding 95% CIs, were calculated for each variable. The optimal cut-off value was determined using the maximum Youden index (J = sensitivity + specificity − 1). Descriptive data are presented as mean ± standard deviation (SD) with 95% CI for normally distributed variables. Continuous variables were expressed as mean ± standard deviation (SD) for normally distributed data and as median (with range) for non-normally distributed data. A two-sided *p*-value < 0.05 was considered statistically significant.

## 3. Results

### 3.1. Characteristics of the Study Sample

Sets of radiographic and echocardiographic images from 93 dogs were included in this study. The mean age of the dogs was 10.48 ± 2.75 years. The study sample comprised 51 males (54.8%) and 42 females (45.2%); 61 dogs (65.6%) were neutered and 32 (34.4%) were intact. The median body weight was 8.8 kg (range, 2.2–44.3 kg). Body weight did not differ significantly between dogs with and without left-sided cardiomegaly (10.39 ± 7.14 kg vs. 14.60 ± 12.23 kg, respectively; *p* = 0.200). Of the 34 breeds the most frequently represented ones were mixed-breeds (*n* = 15, 16.1%), Cavalier King Charles Spaniels (*n* = 13, 14.0%), and Chihuahuas (*n* = 13, 14.0%), followed by Jack Russell Terriers (*n* = 5, 5.4%), Dachshunds (*n* = 4, 4.3%), Border Collies (*n* = 3, 3.2%), and Pomeranians (*n* = 3, 3.2%). The remaining 37 dogs (39.8%) represented other breeds. No classic brachycephalic breeds (such as pug, Boston terrier, Boxer, French bulldog and English bulldog) or sighthounds were included in the study sample.

### 3.2. Echocardiographic Findings

Of the 35 dogs that were classified as stage B1, 5 dogs (14.3%) had isolated left atrial dilation (i.e., equal or exceeding the cut off value), while 2 dogs (5.7%) had isolated left ventricular dilatation. The remaining 28 dogs had both the left atrium as the left ventricle below their respective cut-off values.

Of the 58 dogs with cardiomegaly, 32 dogs were classified as clear stage B2. Classification of the remaining 26 dogs into B2 or C was less straightforward. For establishing stage C disease not only echocardiographic measurements, but also reliable clinical and radiographic information is needed, among other the sleeping respiratory rate, heart rate and rhythm. Because for several dogs these pieces of information were unknown, their classification at the time of echocardiographic examination could not be completed. In addition, some dogs received furosemid prescribed by the referring veterinarian, even if the presence of cardiogenic pulmonary edema had not been confirmed by an increased (i.e., >30 breaths/min) sleeping respiratory rate and radiographs made prior to initiation of the diuretic treatment. Radiographs of dogs with active pulmonary edema were not included in this study.

Detailed echocardiographic findings and between-group comparisons are presented in Table A1.

### 3.3. Radiographic Measurements and Between-Group Comparison

Radiographic long-axis cardiac measurement, short-axis cardiac measurement, and the length of the seventh thoracic vertebra did not differ significantly between dogs with and without echocardiographic left-sided cardiomegaly (*p* = 0.120, *p* = 0.270, and *p* = 0.763, respectively). In contrast, AI-derived vertebral heart score and vertebral left atrial size values were significantly higher in dogs with echocardiographically defined left-sided cardiomegaly than in dogs without cardiomegaly. Mean vertebral heart score was 11.94 ± 1.00 in dogs with cardiomegaly compared with 10.48 ± 1.01 in dogs without cardiomegaly (*p* < 0.001), while mean vertebral left atrial size was 2.75 ± 0.53 for dogs with echocardiographically defined left-sided cardiomegaly and it was 2.28 ± 0.39 for dogs without cardiomegaly (*p* < 0.001).

Similarly, HSVR-T7 was significantly higher in dogs with echocardiographically confirmed cardiomegaly than in dogs without cardiomegaly: 11.65 ± 1.13 versus 10.47 ± 1.08, respectively (*p* < 0.001). Between-group comparisons are presented in Table A2.

### 3.4. Correlation Analysis

**Figure 2:**
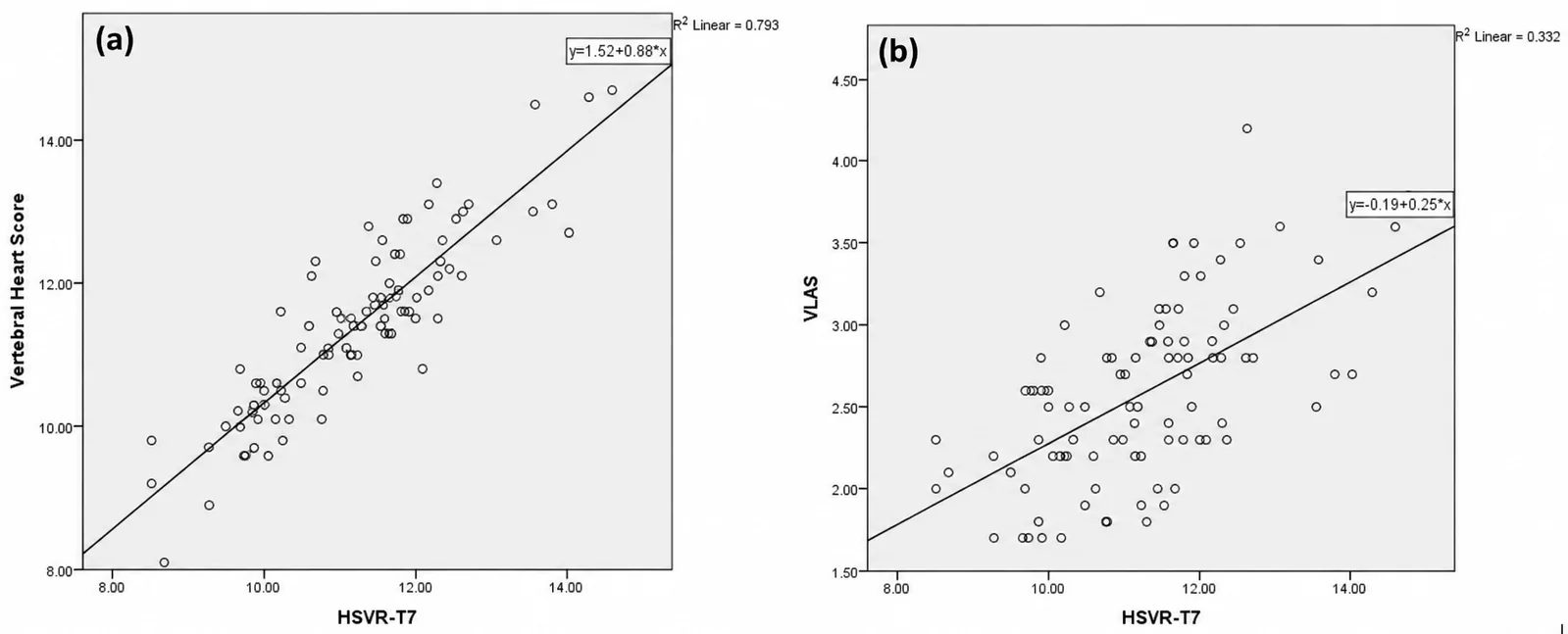
Pearson’s correlation plots showing the relationships between the heart-to-single-vertebra ratio measured at the seventh thoracic vertebra (HSVR-T7) and vertebral heart score (VHS) (**a**) and between HSVR-T7 and VLAS (vertebral left atrial size) (**b**).

Significant positive correlations were identified among all three radiographic measurements (Table 1; Figure 2). The HSVR-T7 showed a very strong positive correlation with vertebral heart score (r = 0.890, *p* < 0.001), and it was moderately positively correlated with vertebral left atrial size (r = 0.576, *p* < 0.001). A strong positive correlation was observed between vertebral heart score and vertebral left atrial size (r = 0.651, *p* < 0.001). The strongest correlation among the evaluated variables was therefore observed between HSVR-T7 and the vertebral heart score.

### 3.5. Diagnostic Performance

**Figure 3:**
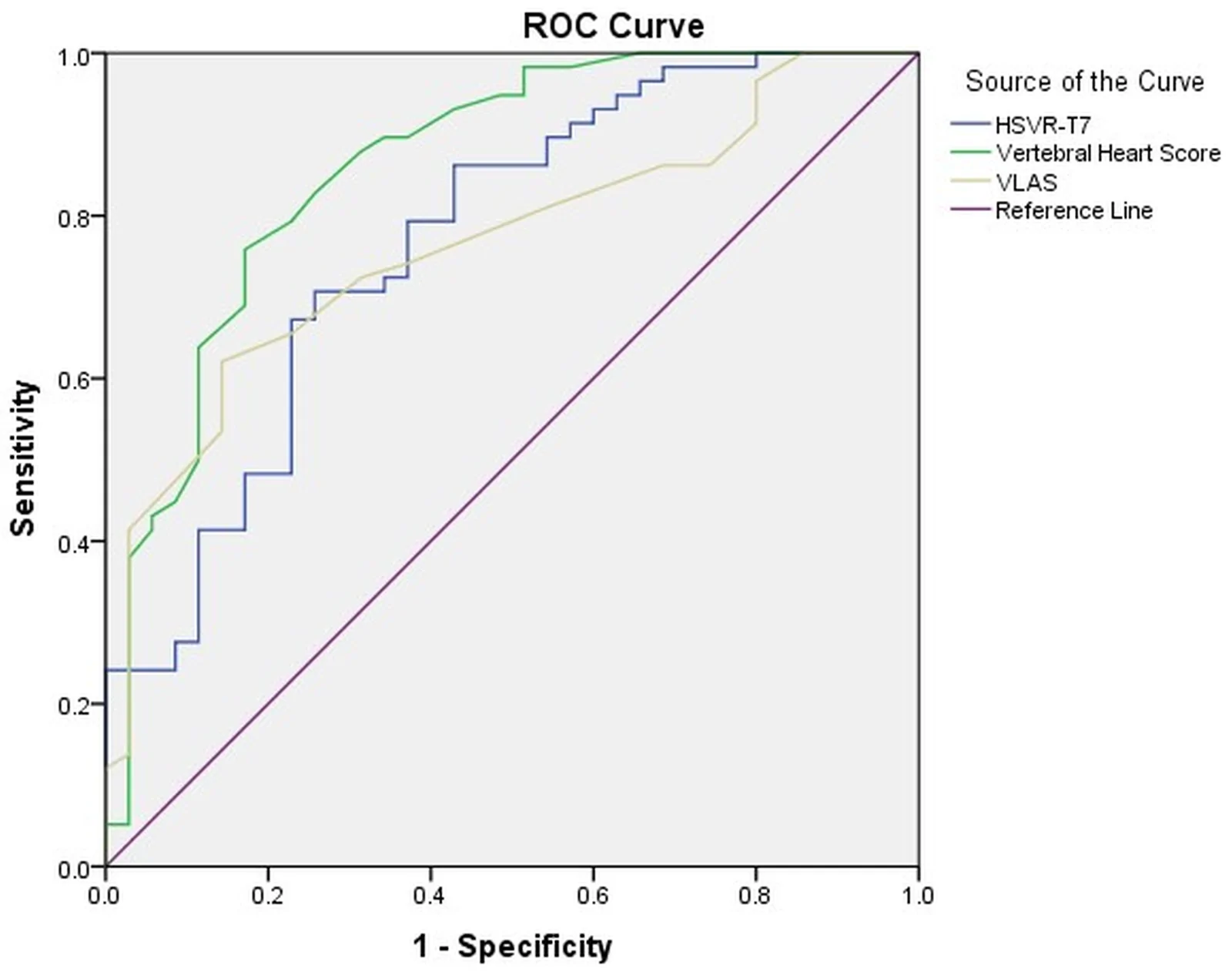
Receiver operating characteristic curves for the HSVR-T7, the AI-derived vertebral heart score, and the AI-derived vertebral left atrial size (VLAS) for discriminating dogs with and without echocardiographically defined left-sided cardiomegaly.

The diagnostic performance of HSVR-T7, AI-derived vertebral heart score, and AI-derived vertebral left atrial size for discriminating dogs with and without echocardiographically defined left-sided cardiomegaly was assessed using ROC curve analysis (Figure 3; Table A3). All three variables demonstrated a significant discriminatory ability (*p* < 0.001).

The HSVR-T7 yielded an AUC of 0.769 (95% CI, 0.669–0.869). At the optimal cut-off value of ≥11.15, the sensitivity was 70.7% and the specificity was 74.3% (Youden J = 0.450). The AI-derived vertebral heart score showed the highest discriminatory performance, with an AUC of 0.861 (95% CI, 0.780–0.942). At the optimal cut-off value of ≥11.35, the vertebral heart score yielded a sensitivity of 75.9%, and a specificity of 82.9% (Youden J = 0.588). The AI-derived vertebral left atrial size yielded an AUC of 0.765 (95% CI, 0.669–0.861), with a sensitivity of 62.1% and specificity of 85.7% at the optimal cut-off value of ≥2.65 (Youden J = 0.478).

Among the three radiographic variables, the AI-derived vertebral heart score demonstrated the highest accuracy. The HSVR-T7 and the vertebral left atrial size showed similar overall discriminatory performance, although HSVR-T7 had higher sensitivity, whereas vertebral left atrial size had higher specificity.

## 4. Discussion

In the present study, we evaluated the diagnostic performance of HSVR-T7 for identifying echocardiographically defined left-sided cardiomegaly in dogs with various stages of myxomatous mitral valve degeneration, and compared the results with AI-derived vertebral heart score and AI-derived vertebral left atrial size measurements. The HSVR-T7 values were higher in dogs with left-sided cardiomegaly, and they showed a very strong positive correlation with the vertebral heart score. However, the hypothesis that the HSVR-T7 would perform better than the vertebral heart score was not supported by our results. These findings suggest that HSVR-T7 may be useful for evaluating cardiomegaly, but it cannot replace echocardiography or vertebral heart score for identifying cardiomegaly.

In the initial description of the method, Costanza et al. (2023) reported that the HSVR-T7 value is consistent with vertebral heart score, and it can serve as an alternative method to vertebral heart score [10]. In our study, the strong correlation between HSVR-T7 and vertebral heart score also supports this finding. However, unlike previous studies, we evaluated not only the relationships of HSVR with vertebral heart score and vertebral left atrial size, but also its ability to distinguish left-sided cardiomegaly and normal-sized hearts as defined by echocardiography. Despite the strong correlation with vertebral heart score, HSVR-T7 showed lower discriminatory performance than the vertebral heart score for detecting cardiomegaly. Therefore, the strong correlation between HSVR-T7 and vertebral heart score does not necessarily indicate that HSVR-T7 has the same diagnostic performance as vertebral heart score for detecting cardiomegaly.

In our study, a very strong correlation was found between HSVR-T7 and vertebral heart score. This may be because both methods use the same cardiac long-axis and short-axis measurements [13]. The main difference between the two methods is that HSVR-T7 is normalized by using the length of a single vertebral body (T7). The use of a single vertebral body length as the denominator may have contributed to the lower discriminatory performance of HSVR-T7, because small biological or measurement-related variations or errors in T7 length might affect the calculated ratio in a greater extent than utilizing a longer reference (i.e., several vertebral bodies). The heterogeneous breed composition and wide body-weight range of our study sample might also have influenced the T7-based ratio. The strong correlation between HSVR-T7 and vertebral heart score may result from their use of the same cardiac measurements and does not indicate that the two methods are completely independent of each other, nor that they are interchangeable.

The absolute long- and short-axis cardiac measurements did not differ significantly between dogs with and without cardiomegaly. This indicates that absolute cardiac measurements alone cannot adequately distinguish between these groups because these measurements are not referenced to body size. In contrast, the vertebral heart score and the HSVR-T7, which adjust the cardiac measurements according to vertebral size, differed significantly between the groups. This finding highlights the importance of vertebral normalization in the radiographic assessment of heart size, similarly as it is done with echocardiographic measurements that are referenced to body weight.

In our study, AI-derived vertebral heart score showed the highest diagnostic performance for detecting cardiomegaly, as defined by echocardiography. The AUC was 0.861, and the optimal cut-off value was 11.35. This cut-off value is very close to the vertebral heart score threshold of ≥ 11.5, as it recommended in the ACVIM consensus statement [1]. Another study reported that vertebral heart score showed moderate diagnostic performance for predicting echocardiographic left-sided heart enlargement in dogs with preclinical myxomatous mitral valve degeneration, with an AUC of 0.851 [14]. Instead of a single optimal cut-off value, these authors proposed two thresholds: vertebral heart score ≤ 10.8 to rule out echocardiographic left-sided heart enlargement, and vertebral heart score > 11.7 to support the presence of cardiac enlargement [14]. The single cut-off value identified in our study falls between these two cut-off values. In another study, the optimal vertebral heart score cut-off value was reported as 11.1 [15], while a review paper reported various vertebral heart score cut-off values of 10.7, 11.0, and 11.5, according to the severity of left atrial enlargement [16].

Recently, artificial intelligence-based software programs for vertebral heart score and vertebral left atrial size measurements have been developed [6,17]. Sykes et al. reported that vertebral heart score and vertebral left atrial size measurements obtained automatically using MetronMind showed good agreement with the measurements that were performed by trained observers [6]. However, in that study, the measurements were not directly compared with echocardiographic diagnosis of cardiomegaly. Also, Gordon et al. evaluated the suitability of another deep learning AI-based application, called RadAnalyzer, for measuring vertebral heart score and vertebral left atrial size in dogs, with similar encouraging results [17]. An important potential advantage of AI-derived measurements is the reduction in observer-dependent variability and human error. However, automated methods do not eliminate measurement errors completely, but seem to increase reproducibility of the measurements [6,17]. Our study evaluated the correlations among AI-derived vertebral heart score, AI-derived vertebral left atrial size, and manually calculated HSVR-T7, as well as the ability of these three measurements to discriminate echocardiographically defined left-sided cardiomegaly. The AI-derived vertebral heart score showed the highest discriminatory performance for detecting cardiomegaly (AUC: 0.861), while AI-derived vertebral left atrial size also showed significant discriminatory performance (AUC: 0.765). These findings suggest that AI-based vertebral heart score and vertebral left atrial size measurements may be useful for the preliminary assessment of cardiomegaly in dogs with myxomatous mitral valve degeneration. Therefore, AI-derived vertebral heart score and vertebral left atrial size may serve as adjunctive radiographic tools for the preliminary assessment of cardiomegaly, particularly when echocardiography is unavailable, but they should not be considered substitutes for echocardiographic evaluation.

In our study, AI-derived vertebral left atrial size showed moderate performance for detecting cardiomegaly defined by echocardiography, with a cut-off value of 2.65 (AUC: 0.765; sensitivity: 62%; specificity: 86%). However, the sensitivity observed in our study was lower than that reported in some of the previous studies. The cut-off value identified in our study is close to the vertebral left atrial size threshold previously reported in dogs with myxomatous mitral valve degeneration. Mikawa et al. reported a cut-off value of 2.6 for identifying stage B2 in dogs with myxomatous mitral valve degeneration, with a sensitivity of 95% and a specificity of 84% [18]. Stepien et al. reported that, for identifying dogs with stage B2 myxomatous mitral valve degeneration, a vertebral left atrial size cut-off of ≥2.5 had a sensitivity of 70% and a specificity of 84%, whereas a vertebral left atrial size cut-off of ≥3.0 had a sensitivity of 40%, and a specificity of 96% [19]. Similarly, Vezzosi et al. reported a sensitivity of 66% and a specificity of 100% for a vertebral left atrial size cut-off value of ≥2.4 in identifying dogs with stage B2 myxomatous mitral valve degeneration [20]. Again another study reported a vertebral left atrial size cut-off value of 2.5 for identifying left-sided cardiomegaly in dogs with myxomatous mitral valve degeneration, with a sensitivity of 69% and a specificity of 97% [21]. One potential reason for the lower diagnostic accuracy of vertebral left atrial size in detecting cardiac enlargement in this study may be that cases with enlargement of only one chamber (either the left atrium or the left ventricle) were categorized as stage B1.

In our study, HSVR-T7 and AI-derived vertebral left atrial size yielded numerically similar AUC values of 0.769 and 0.765, respectively. However, they showed different sensitivity–specificity profiles. The HSVR-T7 showed higher sensitivity (71%), whereas the vertebral left atrial size showed a higher specificity (86%). This difference may be related to the different aspects of cardiomegaly assessed by these methods. While the HSVR-T7 reflects overall cardiomegaly because it is based on the sum of the long- and short-axis cardiac measurements, whereas the vertebral left atrial size specifically evaluates left atrial enlargement. Therefore, the higher sensitivity of the HSVR-T7 may reflect its ability to identify a greater proportion of dogs with generalized cardiomegaly. In contrast, the higher specificity of the vertebral left atrial size suggests that an increased vertebral left atrial size value may more strongly support the presence of left atrial enlargement, although it may fail to identify some dogs with cardiomegaly. The lower AUC values of both methods compared with the AI-derived vertebral heart score measurements suggest that the vertebral heart score may provide a more comprehensive assessment of overall cardiomegaly.

A total of 34 different dog breeds were included in our study. The most common breeds were mixed-breed dogs, Cavalier King Charles Spaniels, and Chihuahuas. Though these latter two breeds do have a brachycephalic skull conformation, they are not typically listed as characteristic brachycephalic breeds, such as the boxer, pug, Boston terrier, and the English and French bulldogs. These latter typical brachycephalic breeds tend to have abnormal (such as hemi-) vertebrae, which might influence the vertebral heart score calculations. Breed-specific reference ranges for the vertebral heart score and the vertebral left atrial size have been reported for several dog breeds [5,22,23,24,25,26,27]. Therefore, it should be considered that the vertebral heart score, vertebral left atrial size, and the HSVR-T7 cut-off values identified in our study may not show the same diagnostic performance across different breeds. Because many breeds were represented by only a few dogs in our study, breed-specific analyses could not be performed.

Several limitations of the present study should be acknowledged. First, dogs with vertebral abnormalities (such as English or French bulldogs) were not included. Without this group of dogs, we cannot assess the intended performance of this new method [10]. Second, the HSVR-T7 was measured by a single observer once; therefore, interobserver variability, and reproducibility of the HSVR-T7 measurement could not be evaluated in the present sample. Also, we have not evaluated intra-observer variability. In addition, the heterogeneous breed composition of our study sample might represent another limitation, but it can also be seen as an advantage. Although breed-specific reference ranges for vertebral heart score and vertebral left atrial size have been reported for several dog breeds [5,22,23,24,25,26,27], our study included 34 different breeds, many of which were represented by only a few dogs. Therefore, breed-specific analyses could not be performed, and the vertebral heart score, vertebral left atrial size, and HSVR-T7 cut-off values identified in our study may not show the same diagnostic performance across different breeds. As the cut-off value for HSVR-T7 was derived from the current study sample, its generalized applicability on dogs with myxomatous mitral valve degeneration should first be validated in another study. Furthermore, dogs with stages B2, and C myxomatous mitral valve degeneration were combined into a single “left-sided cardiomegaly” group. Therefore, the present study evaluated discrimination between echocardiographically defined left-sided cardiomegaly and the absence of cardiomegaly, rather than specifically evaluating how the method can distinguish between dogs with stages B1 and B2 subclinical myxomatous mitral valve degeneration. Finally, only left lateral thoracic radiographs were evaluated, therefore the applicability of the proposed HSVR-T7 cut-off values to right lateral thoracic radiographs remains to be determined. Future studies including dogs with vertebral abnormalities and breed-specific cohorts are warranted to validate the diagnostic performance and proposed cut-off value of HSVR-T7.

## 5. Conclusions

The new method tested, the heart-to-single-vertebral ratio of the 7th thoracic vertebral body (HSVR-T7), provided significantly higher scores in dogs with left-sided cardiomegaly due to myxomatous mitral valve degeneration (stages B2 and C) than in dogs without cardiomegaly (stage B1). Though HSVR-T7 showed a strong positive correlation with vertebral heart score, its diagnostic accuracy for identifying cardiomegaly in dogs with myxomatous mitral valve degeneration is only moderate. The AI-based vertebral heart score calculation showed the highest diagnostic performance among the three radiographic methods to distinguish dogs with cardiomegaly and normal-sized hearts.

## Figures and Tables

**Table 1:** Pearson’s correlation coefficients among vertebral heart score (VHS), vertebral left atrial size (VLAS), and heart-to-single-vertebra ratio measured at the seventh thoracic vertebra (HSVR-T7). ** *p* < 0.001.

Variable	VHS	VLAS	HSVR-T7
VHS	1	0.651 **	0.890 **
VLAS	0.651 **	1	0.576 **
HSVR-T7	0.890 **	0.576 **	1

## Data Availability

The original contributions presented in this study are included in this article. Further inquiries can be directed to the corresponding authors.

## Appendix A

**Table A1:** Body weight and echocardiographic measurements according to cardiomegaly status of 93 dogs with myxomatous mitral valve degeneration. Superscript letters (a, b) within the same row indicate a statistically significant difference between groups (*p* < 0.05). LA/Ao, left atrial-to-aortic ratio; LVIDd, left ventricular internal diameter in diastole; LVIDdN, body-weight-normalized.

Variable	No Cardiomegaly (*n* = 35) Mean ± SD (95% CI)	Cardiomegaly (*n* = 58) mean ± SD (95% CI)	Mann–Whitney U	*p* Value
LA/Ao	1.48 ± 0.34 (95% CI: 1.35–1.60) ^a^	2.46 ± 0.75 (95% CI: 2.25–2.66) ^b^	125.0	<0.001
LVIDd	33.77 ± 9.43 (95% CI: 30.43–37.12) ^a^	40.33 ± 12.65 (95% CI: 36.97–43.68) ^b^	626.0	0.008
LVIDdN	1.61 ± 0.26 (95% CI: 1.52–1.70) ^a^	2.14 ± 0.33 (95% CI: 2.05–2.23) ^b^	194.5	<0.001

**Table A2:** Radiographic measurements according to cardiomegaly status of 93 dogs with myxomatous mitral valve degeneration. Different superscript letters (a, b) within the same row indicate a statistically significant difference between groups (*p* < 0.05).

Parameter	No Cardiomegaly (*n* = 35) Mean ± SD (95% CI)	Cardiomegaly (*n* = 58) Mean ± SD (95% CI)	*p* Value
Long axis (mm)	87.41 ± 28.91 (77.48–97.34)	94.36 ± 26.98 (87.27–101.46)	0.120
Short axis (mm)	79.15 ± 21.92 (71.62–86.68)	84.78 ± 24.79 (78.26–91.30)	0.270
7th thoracic vertebra (mm)	15.97 ± 4.87 (14.30–17.65)	15.32 ± 4.03 (14.26–16.38)	0.763
Vertebral Heart Score	10.48 ± 1.01 ^a^ (10.13–10.82)	11.94 ± 1.00 ^b^ (11.68–12.20)	<0.001
Vertebral Left Atrial Size	2.28 ± 0.39 ^a^ (2.15–2.41)	2.75 ± 0.53 ^b^ (2.61–2.89)	<0.001
HSVR-T7	10.47 ± 1.08 ^a^ (10.11–10.84)	11.65 ± 1.13 ^b^ (11.36–11.95)	<0.001

**Table A3:** ROC curve analysis and optimal cut-off values for identifying echocardiographically defined cardiomegaly for the three radiographic measurements.

Variable	AUC (95% CI)	SE	*p* Value	Optimal Cut-Off *	Sensitivity (95% CI)	Specificity (95% CI)	Youden J
HSVR-T7	0.769 (0.669–0.869)	0.051	<0.001	≥11.15	70.7% (57.3–81.9)	74.3% (56.7–87.5)	0.450
VHS	0.861 (0.780–0.942)	0.041	<0.001	≥11.35	75.9% (62.8–86.1)	82.9% (66.4–93.4)	0.588
VLAS	0.765 (0.669–0.861)	0.049	<0.001	≥2.65	62.1% (48.4–74.5)	85.7% (69.7–95.2)	0.478

* The optimal cut-off values were determined using the Youden index (sensitivity + specificity − 1).
